# Interstitial Pneumonia with Autoimmune Features from the Rheumatologists’ Perspective; Single Center Experience

**DOI:** 10.3390/diagnostics16020299

**Published:** 2026-01-17

**Authors:** Emine Uslu, Didem Sahin, Ahmet Ilbay, Recep Yilmaz, Abdulbaki Gaydan, Nilgun Govec Giynas, Ahmet Usta, Yeter Mahmutoglu, Rahime Aksoy, Serdar Sezer, Mucteba Enes Yayla, Melahat Kul, Aysegul Gursoy Coruh, Caglar Uzun, Ebru Us, Ozlem Ozdemir Kumbasar, Askin Ates, Tahsin Murat Turgay

**Affiliations:** 1 Division of Rheumatology, Department of Internal Medicine, Faculty of Medicine, Ankara University, Ankara 06230, Turkey; dr.didemsa@gmail.com (D.S.); ilbayahmet@gmail.com (A.I.); recep.yilmaz0621@gmail.com (R.Y.); abdulbaki234@gmail.com (A.G.); drnggiynas@gmail.com (N.G.G.); ahmet.2254@hotmail.com (A.U.); dr.yetermahmutoglu@gmail.com (Y.M.); serdarsezer1987@hotmail.com (S.S.); enesyayla@hotmail.com (M.E.Y.); askinates1970@hotmail.com (A.A.); tmturgay@hotmail.com (T.M.T.); 2 Department of Hematology, Faculty of Medicine, Ankara University, Ankara 06230, Turkey; rahimeaksoy77@yahoo.com; 3 Department of Radiology, Faculty of Medicine, Ankara University, Ankara 06230, Turkey; melahatkul@yahoo.com (M.K.); draysegulgursoy@gmail.com (A.G.C.); cuzun77@yahoo.com (C.U.); 4 Department of Medical Microbiology, Faculty of Medicine, Ankara University, Ankara 06230, Turkey; eus@medicine.ankara.edu.tr; 5 Department of Pulmonology, Faculty of Medicine, Ankara University, Ankara 06620, Turkey; ozlemozdemir@yahoo.com

**Keywords:** interstitial lung disease, autoimmunity, cyclophosphamide, glucocorticoids

## Abstract

**Background/Objectives**: Interstitial pneumonia with autoimmune features (IPAF) is a recently defined entity characterized by interstitial lung disease (ILD) with clinical, serological, and radiological features suggestive of autoimmunity that do not fulfil the criteria for a defined connective tissue disease (CTD). This study aimed to evaluate the clinical characteristics, treatment modalities, and outcomes of patients with IPAF in a tertiary referral center. **Methods**: We retrospectively analyzed 72 patients who fulfilled the IPAF classification criteria. Demographic, clinical, serological, radiological, pulmonary function, treatment, and survival data were collected and evaluated. Logistic regression analysis was performed to identify factors associated with mortality. **Results**: The cohort consisted of 62.5% female patients, with a mean age of 62.7 (SD, 10.4) years at diagnosis. The most frequent radiological pattern was nonspecific interstitial pneumonia (83.3%). Raynaud’s phenomenon (6.9%) and arthritis (2.8%) were the most common rheumatological manifestations. Antinuclear antibodies positivity at titers ≥1:320 was observed in 27.8% of patients. Azathioprine was the most frequently prescribed agent (20.8%), followed by mycophenolate mofetil (11.1%). After a median follow-up of 30.1 months (IQR, 52.8), 16 patients (22.22%) died, with a 5-year survival rate of 70%. Glucocorticoid therapy at doses ≥20 mg/day was independently associated with increased mortality (OR 6.13 (95% CI 1.17–32.21). **Conclusions**: IPAF predominantly affects middle-aged females. Glucocorticoid use at doses ≥20 mg/day was associated with mortality; however, this observational association may reflect underlying disease severity rather than a causal effect of high-dose treatment. Further prospective studies are needed to optimize management strategies in patients with IPAF.

## 1. Introduction

Interstitial lung disease (ILD) refers to a group of disorders characterized by impaired gas exchange resulting from inflammation and fibrosis of the lung interstitium [1]. ILD can be classified into several categories, including idiopathic, autoimmune-related, exposure-related, sarcoidosis, and eosinophilic lung diseases [1,2]. Patterns of ILD involvement vary according to age and sex. Idiopathic pulmonary fibrosis (IPF) predominantly affects older men, whereas connective tissue disease–related ILD (CTD-ILD) is more common among middle-aged women [3,4,5]. Despite established classification systems, a subset of ILD patients remains unclassified.

The term ‘interstitial pneumonia with autoimmune features’ (IPAF) is a relatively recent concept, first proposed in 2015 by the European Respiratory Society and American Thoracic Society (ERS/ATS). IPAF refers to patients who exhibit a combination of serological, clinical, and radiological features suggestive of connective tissue diseases (CTDs), yet do not fulfil the established classification criteria for any specific CTD [6].

IPAF affects a population that is generally younger than those with IPF but older than patients with CTD-ILD. It tends to occur slightly more frequently in females than in males [7]. The survival of patients with IPAF is poorer than that of CTD-ILD patients, but better than that of patients with IPF [7].

Although IPAF has been defined as a distinct entity, there are no clear recommendations regarding treatment modalities due to the heterogeneous nature of the disease and its still unclear underlying etiopathogenesis. Treatment choices and timing in IPAF patients remain unclear.

The IPAF is a relatively newly defined disease, and data are limited on the most affected age group and sex, presenting symptoms, predominant patterns of lung involvement, common serological findings, and overall life expectancy associated with the disease. The aim of our study is to describe the clinical, demographic, and laboratory characteristics of patients diagnosed with IPAF at our center and to retrospectively evaluate factors associated with survival.

## 2. Materials and Methods

The medical records of 386 consecutive patients evaluated for interstitial lung disease (ILD) who presented to the inpatient and outpatient rheumatology clinics at Ankara University between 2015 and 2023 were retrospectively analyzed. All patients diagnosed with interstitial lung disease were evaluated regardless of possible etiology. Of the 386 patients, those meeting the 2015 ERS/ATS classification criteria for autoimmune-associated interstitial pneumonia (IPAF) were identified and included in the study. A total of 304 patients (78.8%) were excluded from the study because they met a CTD classification criterion, an IPF classification criterion, or were classified as hypersensitivity pneumonitis. Of the 82 patients (21.2%) who met the 2015 IPAF classification criteria, 10 were excluded from the analyses because they also fulfilled the 2019 EUSTAR-VEDOSS criteria (1 patient) and 2017 ACR/EULAR idiopathic inflammatory myopathies (IIM) criteria (9 patients) [6,8,9]. The final cohort consisted of 72 patients (18.6%) who met the ERS/ATS criteria for IPAF and were therefore included in the study [6].

Two independent radiologists (AC and MK), who were blinded to the clinical data of the included patients, re-evaluated the chest computed tomography scans (chest-CT). In cases of disagreement, a third radiologist (CU) was consulted, and the three radiologists discussed the findings until a consensus was reached. The chest-CT findings were classified using the definitions from the Idiopathic Pulmonary Fibrosis and Progressive Pulmonary Fibrosis in Adults: An Official ATS/ERS/JRS/ALAT Clinical Practice Guideline [2,10]. The reticular pattern associated with honeycombing, with or without traction bronchiectasis, primarily located in the subpleural regions and lung bases, was classified as usual interstitial pneumonia (UIP). The organising pneumonia (OP) pattern was defined by bilateral patchy areas of consolidation and/or ground-glass opacities with subpleural and inferior lung predominance or peribronchovascular distribution. The nonspecific interstitial pneumonia (NSIP) pattern was defined as basal predominant reticular abnormalities with traction bronchiectasis associated with ground-glass attenuation with preservation of the subpleural space. Diffuse involvement without subpleural predominance, which did not suggest any specific features of pulmonary fibrosis, was classified as indeterminate [2,10]. In patients who underwent lung biopsy, the histopathological pattern was considered the primary determinant of lung involvement.

Data on age at IPAF diagnosis, laboratory and serological characteristics, rheumatological symptoms and findings, comorbidities, treatment modalities, and pulmonary function tests (PFTs) were recorded from the patients’ files. Glucocorticoid exposure was defined based on the highest daily dose of oral prednisolone received for at least one consecutive month at any time during follow-up. Patients were categorized into three groups: no oral glucocorticoid use for an IPAF indication during follow-up (Group 1), low-dose exposure (>0–19 mg/day; Group 2), and high-dose exposure (≥20 mg/day; Group 3). Patients who received pulse glucocorticoid therapy were subsequently treated with oral glucocorticoids and were classified according to their sustained oral dose. The date of IPAF diagnosis was defined as the date of the first chest-CT scan demonstrating interstitial lung involvement.

Baseline PFTs and diffusion capacity for carbon monoxide (DLCO) test results were also noted. Forced vital capacity (FVC) values in baseline PFTs were divided into three groups: patients with FVC values ≥ 80%, those with values between 79% and 60%, and those with values below 60%. Patients were also categorized according to DLCO values as ≥80%, between 60–79%, between 40–59%, and below 40%.

Hypoxemia was defined as a baseline arterial PaO_2_ < 60 mmHg on arterial blood gas analysis.

The follow-up period was defined as the time from the patient’s first hospital admission to their last admission. Survival time was calculated from the date of first admission to the date of death or last known follow-up, based on the vital status (alive or deceased) recorded in the hospital system.

Ethical approval was obtained from the Ethics Committee of Ankara University Faculty of Medicine, numbered İ10-820-24.

### Statistical Analyses

Data analysis was performed by using IBM SPSS (Statistical Package for Social Sciences) version 26 package program. Visual (histogram and probability graphs) and analytical methods (Kolmogorov-Smirnov/Shapiro-Wilk tests) were used to determine whether variables conformed to the normal distribution. Descriptive analyses were reported as median and interquartile range (IQR) for numerical variables that were not normally distributed, and as frequency tables for ordinal and categorical variables.

For group comparisons, the Student’s *t*-test was used for normally distributed numerical variables, and the Mann-Whitney U test for those not normally distributed. Categorical variables were analysed using the Chi-square test or Fisher’s exact test, as appropriate. Logistic regression analyses were performed to identify independent predictors associated with all-cause mortality. Variables with *p*-value < 0.10 in the univariate analyses were included in the multivariate logistic regression model. The multivariate model was additionally adjusted for sex, age at diagnosis, and smoking status. Smoking status was included irrespective of its univariate significance because it is a well-established factor associated with mortality in the literature [11]. Hosmer-Lemeshow test was used for model fit. Results were considered statistically significant for *p* < 0.05. Life-table analysis and Kaplan–Meier analysis were used to conduct survival analyses.

## 3. Results

Of 72 patients who fulfilled the ERS/ATS criteria for IPAF, 45 (62.5%) were female. The mean age at diagnosis was 62.7 (SD: 10.4) years and the median follow-up period was 30.1 months (IQR, 52.8). Symptom duration was available for 71 patients in the overall cohort and showed a mean duration of 2.3 ± 2.5 years. Thirty patients (41.7%) had a history of smoking. Baseline body mass index (BMI) data were available for 62 patients. The mean BMI in the overall cohort was 29.5 ± 4.5 kg/m^2^. Arterial blood gas analysis data were available for 51 patients. Hypoxemia was observed in 14 patients (27.5%) overall, including 11 patients with NSIP (25.6%), 2 with OP (33.3%), and 1 with an indeterminate pattern (50.0%) (Table 1). Respiratory symptoms were present in 70 patients (97.2%). The most common symptoms were dyspnea and cough (91.7% and 83.3%, respectively). The most common rheumatological findings were Raynaud’s phenomenon and arthritis (6.9% and 2.8%, respectively) (Table 2). Digital ulcers, palmar telangiectasia, puffy edema, mechanic hands, and Gottron’s sign were not observed in any of the patients.

Following radiological and pathological assessment, the most common pattern of pulmonary involvement was NSIP, observed in 60 (83.3%) patients, followed by OP in 8 (11.1%) patients, and indeterminate pattern in 4 (5.6%). A UIP pattern was not observed in our study population. The demographic and clinical characteristics of the patients based on lung involvement patterns are shown in Table 1. Five patients underwent lung biopsy, among whom two were consistent with NSIP and three with OP.

The serological profiles of patients with IPAF are presented in Table 3. Antinuclear antibody (ANA) titers ≥ 1:320 (diffuse, speckled, or homogeneous patterns) were positive in 20 patients (27.8%), whereas ANA with nucleolar or centromere patterns was positive in 16 patients (22.2%). A positive rheumatoid factor (RF) at levels of at least twice the upper limit of normal (ULN) was detected in 16 patients (23.2%), followed by anti-cyclic citrullinated peptide (anti-CCP) antibody positivity in 12 patients (19.4%) (Table 3).

Baseline FVC measurements were available for 54 patients. Of these, 40.7% (*n* = 22) had FVC values ≥ 80% of the predicted value, 35.2% (*n* = 19) had values between 60% and 79%, and 24.1% (*n* = 13) had FVC values < 60%. Among the 47 patients with available DLCO measurements, 3 patients (6.4%) had DLCO values ≥ 80% of the predicted value, 9 patients (19.1%) had values between 60% and 79%, 15 patients (31.9%) had values between 40% and 59%, and 20 patients (42.6%) had DLCO values < 40%.

Immunosuppressive treatment was initiated in a total of 26 patients (36.1%), with azathioprine (AZA) being the most frequently used agent in 15 patients (20.8%), followed by mycophenolate mofetil (MMF), which was administered to 8 patients (11.1%) (Table 4). Additionally, 3 patients (4.2%) received cyclophosphamide (CYC) treatment, and 2 patients (2.8%) received rituximab. No deaths were observed among patients who received cyclophosphamide. Only two patients (2.7%) underwent multiple cycles of immunosuppressive therapy. In one patient, toxic hepatitis developed during AZA treatment, necessitating CYC therapy; however, the patient was lost to follow-up after the sixth cycle of CYC. In the other patient, disease reactivation during AZA treatment prompted induction therapy with CYC, followed by maintenance therapy with MMF. Three patients (4.2%) received antifibrotic treatment. Among them, one patient received an antifibrotic agent together with AZA and glucocorticoids, while the other two received antifibrotic therapy in combination with glucocorticoids only. Thirty-four patients (47.2%) received ≥20 mg/day of glucocorticoids, whereas 15 patients (20.8%) received <20 mg/day. In total, 3 (4.2%) patients received pulse glucocorticoid treatment, and 2 of these patients died. One death was due to an Acinetobacter infection, and the other was due to a cytomegalovirus infection. All deaths occurred within the first month after diagnosis. The maximum daily doses of glucocorticoid treatment across the cohort were reported in Table 4. Of the total cohort, 17 patients (23.6%) did not receive any immunosuppressive therapy, glucocorticoids, or antifibrotic agents during their follow-up.

Of the 72 patients, 27 (37.5%) required long-term oxygen therapy (LTOT); of these, 17 (63.0%) started LTOT during follow-up, while 10 (37.0%) started at the time of diagnosis or within the preceding 6 months. Among the 17 patients who initiated LTOT during follow-up, the median time to LTOT initiation was 18.0 months (IQR, 44.0).

During the follow-up period, definitive CTD was diagnosed in 1 patient (1.39%), who progressed to rheumatoid arthritis 13 months after the initial diagnosis of IPAF. At the time of IPAF onset, the patient had no clinical arthritis, was RF-positive and anti-CCP negative, and exhibited an NSIP pattern on chest-CT.

**Figure 1:**
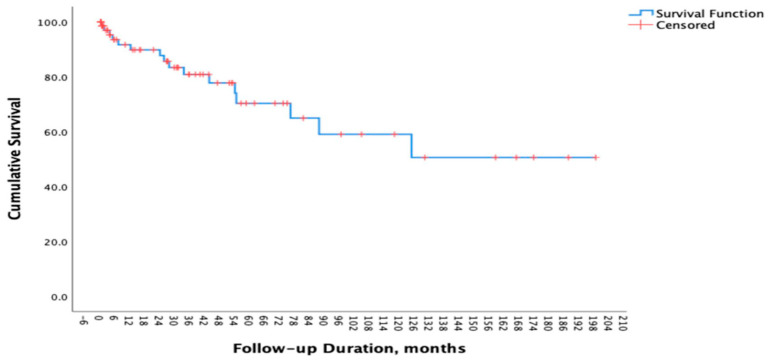
Survival Curve of Patients with IPAF.

After a median follow up of 30.1 (IQR: 52.9) months, 16 patients (22.22%) died. The 5-year survival rate in our cohort was 70% (Figure 1). Of 16 patients, 3 patients (18.75%) died of respiratory failure; 3 patients (18.75%) died due to infections (Acinetobacter infection, cytomegalovirus pneumonia, and COVID-19 pneumonia) and 1 patient (6.25%) died of non-small cell lung cancer. The causes of death for the remaining 9 individuals could not be determined. In our cohort, smoking history, comorbidities, autoantibody status, sex, and lung involvement patterns were not associated with mortality. No significant differences were observed between deceased and surviving patients with respect to symptom duration (2.0 ± 1.9 vs. 2.4 ± 2.6 years, *p* = 0.615), BMI (28.0 ± 4.9 vs. 29.8 ± 4.4 kg/m^2^, *p* = 0.232), or the presence of hypoxemia (41.7% vs. 23.1%, *p* = 0.272). The differences in the characteristics of deceased and surviving patients are summarized in Supplementary Table S1. In univariable logistic regression analysis, daily oral glucocorticoid use at doses ≥20 mg/day was associated with an increased risk of mortality (OR 5.02, 95% CI 0.99–25.34; *p* = 0.051). In the multivariable model adjusted for age, sex, and smoking status, glucocorticoid use ≥20 mg/day remained independently associated with mortality (OR 6.13, 95% CI 1.17–32.21; *p* = 0.032) (Table 5).

## 4. Discussion

In our study, IPAF predominantly affected patients around 60 years of age and was relatively more common in women. No specific autoantibody, clinical symptom, or finding associated with increased disease-related mortality was identified. However, mortality was higher in patients receiving glucocorticoid therapy equivalent to 20 mg or more of prednisolone.

Of the 82 patients initially fulfilling the 2015 ERS/ATS criteria for IPAF, 10 patients (12.2%) were subsequently excluded as they also met newer CTD classification criteria (EUSTAR-VEDOSS, ACR/EULAR IIM). This finding highlights the limitation of the 2015 ERS/ATS definition: patients classified as IPAF according to these criteria may, in fact, be reclassified as having a defined CTD when more up-to-date rheumatological criteria are applied. Such discrepancies (12.2% in our cohort) highlight the need for updated multidisciplinary IPAF classification criteria that are compatible with current CTD definitions.

CTD-ILD patients are known to be younger, predominantly female and non-smokers [12]. In contrast, the median age at diagnosis of IPF is 66 years, with prevalence increasing with age. IPF predominantly affects men and smokers [13,14]. According to Öz et al., while male sex predominated in IPF patients and female sex in CTD-ILD patients, IPAF patients showed a female predominance, though to a lesser extent than CTD-ILD [15]. IPAF studies have shown that the median age at diagnosis is approximately 60–63 years, with a female preponderance. In our study, the mean age at diagnosis and sex distribution were similar to those reported in previous studies [7,16]. This finding suggests that IPAF patients resemble those with IPF in terms of age but differ in terms of sex distribution. Oldham et al. reported that 54% of IPAF patients had a history of smoking at some point in their lives [7]. Among patients with CTD-ILD, the proportion of individuals with any history of smoking has been reported to range between 31% and 43% [17,18]. In contrast, 73–84% of patients with IPF have a history of smoking [19,20]. In our cohort, 41.7% of patients had a history of smoking.

In line with previous studies, Raynaud’s phenomenon and arthritis were the most common clinical manifestations in our cohort [7,21,22]. Consistent with earlier findings, the presence of Raynaud’s phenomenon was not associated with mortality [22]. Similar to previous reports, no digital ulcers were observed in our patients [21,22]. Previous studies on palmar telangiectasia have reported variable frequencies, ranging from absence to up to 25%; however, none of the patients in our study exhibited this finding [7,21,22]. In contrast to earlier research, puffy oedema and mechanic’s hands were not observed in any of the patients in our cohort [7,21,22].

While NSIP has been reported as the most common radiological pattern in IPAF patients in previous studies, some have also suggested that its presence may be associated with a poorer prognosis [7,23]. In our cohort, NSIP was likewise the most frequently observed pattern; however, it was not found to be associated with increased mortality.

The 5-year survival rate in patients with IPF has been estimated to range between 45% and 53% [24,25,26]. In our cohort, the 5-year survival rate was 70%, exceeding the expected rate for patients with IPF. Previous studies have identified age, FVC, and partial pressure of oxygen as independent prognostic indicators in IPAF [27,28]. Moreover, smoking history and anti-RNP antibody positivity have been linked to poorer outcomes [29]. After adjustment for age, sex, and smoking status, treatment with glucocorticoids at doses ≥20 mg/day was independently associated with mortality. However, the possibility that higher glucocorticoid doses may be preferred in patients with severe or rapidly progressing disease should also be considered. Furthermore, infections that may develop in association with immunosuppression should not be overlooked. Therefore, individualized treatment decisions should be made and outcomes should be carefully interpreted in patients receiving high-dose glucocorticoids.

Approximately one-third of patients in our cohort did not receive immunosuppressive therapy. This may be related to the lack of clearly defined treatment strategies for IPAF and the absence of obvious systemic autoimmune symptoms in many patients. This may have led clinicians to avoid immunosuppressive therapy due to concerns about infection risk and treatment-related side effects. Antifibrotic therapy has rarely been used in our patients. This may be related to the fact that antifibrotic therapy in IPAF patients is not covered by health authorities. Furthermore, the lack of data supporting routine antifibrotic use in IPAF may also contribute to this situation.

Our study has certain limitations due to its retrospective, single-center design, including a relatively small sample size and missing data. The outcomes of patients who did not attend follow-up appointments remain unknown, and the severity of the disease cannot be fully determined. Nevertheless, we tried to mitigate these limitations by re-evaluating chest CT scans, which were assessed by experienced radiologists, and thoroughly reviewing the patients’ medical records. One of the major limitations of this study is the incomplete availability of baseline PFT data. Therefore, although PFT results may have a significant effect on mortality, they could not be included in multivariate analyses.

## 5. Conclusions

In our study, IPAF was slightly more common in females, with diagnosis typically occurring at approximately 60 years of age. Autoantibodies, chest-CT patterns, and sex were not found to be associated with mortality. Mortality was higher among patients who received high-dose glucocorticoid therapy. This finding should be interpreted cautiously, as it does not establish a causal relationship and may reflect underlying disease severity rather than a direct harmful effect of treatment. Overall, these results underscore the need for careful clinical decision-making and further prospective studies to better define prognostic factors and optimize treatment strategies in patients with IPAF.

## Figures and Tables

**Table 1:** Demographic, clinical and laboratory characteristics of the patients based on the lung involvement.

	All *n* = 72	NSIP *n* = 60	OP *n* = 8	Indeterminate *n* = 4
Female sex, *n* (%)	45 (62.5)	37 (61.7)	5 (62.5)	3 (75)
Age at diagnosis, year, mean (SD)	62.7 (10.4)	63.1 (9.9)	56.5 (13.4)	68.9 (5.9)
Symptom Duration, years, mean (SD)	2.3 (2.5)	2.5 (2.6)	1.6 (1.8)	0.6 (0.5)
Smoking, ever, *n* (%)	30 (41.7)	25 (41.7)	3 (37.5)	2 (50.0)
BMI, mean (SD)	29.5 (4.5)	29.3 (4.3)	30.0 (6.6)	32.2 (2.5)
CRP				
Normal, *n* (%)	31 (41.3)	24 (40.0)	3 (37.5)	4 (100.0)
>2 × ULN, *n* (%)	26 (36.1)	22 (36.7)	4 (50.0)	0 (0.0)
ESR, median (IQR)	16 (27.0)	16 (26.0)	22 (70.0)	11 (21.0)
Comorbidities				
Diabetes mellitus, *n* (%)	19 (26.4)	17 (28.3)	1 (12.5)	1 (25.0)
Hypertension, *n* (%)	30 (41.7)	26 (43.3)	3 (37.5)	1 (25.0)
Chronic lung disease, *n* (%)	10 (13.9)	7 (11.7)	1 (12.5)	2 (50.0)
Atherosclerotic heart disease, *n* (%)	16 (22.2)	13 (21.7)	1 (12.5)	2 (50.0)
Malignancy, *n* (%)	6 (8.3)	5 (8.3)	1 (12.5)	0 (0.0)
LTOT, *n* (%)	27 (37.5)	23 (38.3)	3 (37.5)	1 (25.0)
Hypoxemia, *n* (%)	14 (27.5)	11 (25.6)	2 (33.3)	1 (50.0)
Death, *n* (%)	16 (22.2)	14 (23.3)	1 (12.5)	1 (25.0)

NSIP: Nonspecific interstitial pneumonia. OP: Organising pneumonia. CRP: C-Reactive Protein. ULN: Upper Limit of Normal. ESR: Erythrocyte sedimentation rate, LTOT: Long-Term Oxygen Therapy, BMI: Body mass index.

**Table 2:** Symptoms and findings of IPAF patients.

	All *n* = 72	NSIP *n* = 60	OP *n* = 8	Indeterminate *n* = 4
Any respiratory symptom, *n* (%)	70 (97.2)	58 (96.7)	8 (100.0)	4 (100.0)
Dyspnea, *n* (%)	66 (91.7)	55 (91.7)	7 (87.5)	4 (100.0)
Cough, *n* (%)	60 (83.3)	49 (81.7)	8 (100.0)	3 (75.0)
Chest Pain, *n* (%)	14 (19.4)	11 (18.3)	2 (25.0)	1 (25.0)
Sputum, *n* (%)	24 (33.3)	18 (30.0)	5 (62.5)	1 (25.0)
Ral, *n* (%)	55 (76.4)	46 (76.7)	7 (87.5)	2 (50.0)
Arthritis, *n* (%)	2 (2.8)	2 (3.3)	0 (0.0)	0 (0.0)
Morning stiffness ≥ 60 min, *n* (%)	4 (5.6)	4 (6.7)	0 (0.0)	0 (0.0)
Raynaud’s phenomenon, *n* (%)	5 (6.9)	3 (5.0)	0 (0.0)	2 (50.0)
Gottron’s sign, *n* (%)	0 (0.0)	0 (0.0)	0 (0.0)	0 (0.0)

NSIP: Nonspecific interstitial pneumonia. OP: Organising pneumonia.

**Table 3:** Serological characteristics of IPAF patients.

	All *n* = 72	NSIP *n* = 60	OP *n* = 8	Indeterminate *n* = 4
Low positive ANA, *n* (%)	21 (29.2)	17/60 (28.3)	2/8 (25.0)	2 (50.0)
ANA ≥1:320, *n* (%) (diffuse, speckled, homogeneous)	20 (27.8)	19/60 (31.7)	0/8 (0)	1 (25.0)
ANA positivity, *n* (%) (nucleolar or centromere),	16 (22.2)	15/60 (25.0)	1/8 (12.5)	0 (0.0)
RF ≥ 2ULN, *n* (%)	16/69 (23.2)	13/58 (22.4)	3/7 (42.9)	0/4 (0.0)
Anti-CCP > ULN, *n* (%)	12/62 (19.4)	12/53 (22.6)	0/6 (0)	0/3 (0.0)
Anti-dsDNA, *n* (%)	7/59 (11.9)	5/48 (10.4)	1/7 (14.3)	0/3 (0.0)
Anti-Ro (SS-A), *n* (%)	5/69 (7.2)	5/58 (8.6)	0/7 (0.0)	0/4 (0.0)
Anti-La (SS-B), *n* (%)	5/69 (7.2)	4/58 (6.9)	0/7 (0.0)	1/4 (25.0)
Anti-Ro-52, *n* (%)	5/69 (7.2)	5/58 (8.6)	0/7 (0.0)	0/4 (0.0)
Anti-Smith, *n* (%)	2/69 (2.9)	1/58 (1.7)	1/7 (14.3)	0/4 (0.0)
Anti-Jo-1, *n* (%)	0/69 (0.0)	0/58 (0.0)	0/7 (0.0)	0/4 (0.0)
Anti-Scl-70, *n* (%)	0/69 (0.0)	0/58 (0.0)	0/7 (0.0)	0/4 (0.0)
Anti-PM-Scl, *n* (%)	4/66 (6.1)	3/55 (5.5)	0/7 (0.0)	1/4 (25.0)

NSIP: Nonspecific interstitial pneumonia. OP: Organising pneumonia. ANA: Anti-nuclear antibody, RF: Rheumatoid factor. ULN: Upper Limit of Normal. Anti-CCP: Anti-cyclic citrullinated peptides.

**Table 4:** Treatment characteristics of the study population.

	All *n* = 72	NSIP *n* = 60	OP *n* = 8	Indeterminate *n* = 4
Pulse GCs, ever, *n* (%)	3 (4.2)	3 (5.0)	0 (0.0)	0 (0.0)
Oral GCs, ever, *n* (%)	49 (68.1)	40 (66.7)	8 (100.0)	1 (25.0)
Daily Oral GCs dose				
Never	23 (31.9)	20 (33.3)	0 (0.0)	3 (75.0)
>0–19 mg/day	15 (20.8)	12 (20.0)	3 (37.5)	0 (0.0)
≥20 mg/day	34 (47.2)	28 (46.7)	5 (62.5)	1 (25.0)
IS without GCs, *n* (%)	6 (8.3)	6 (10.0)	0 (0.0)	0 (0.0)
Cyclophosphamide, *n* (%)	3 (4.2)	3 (5.0)	0 (0.0)	0 (0.0)
Methotrexate, *n* (%)	3 (4.2)	2 (3.3)	1 (12.5)	0 (0.0)
Hydroxychloroquine, *n* (%)	6 (8.3)	4 (6.7)	1 (12.5)	1 (25.0)
Rituximab, *n* (%)	2 (2.8)	1 (1.7)	1 (12.5)	0 (0.0)
MMF, *n* (%)	8 (11.1)	7 (11.7)	1 (12.5)	0 (0.0)
Azathioprine, *n* (%)	15 (20.8)	14 (23.3)	1 (12.5)	0 (0.0)
Nintedanib/Pirfenidone, *n* (%)	3 (4.2)	2 (3.3)	1 (12.5)	0 (0.0)
No treatment, *n* (%)	17 (23.6)	14 (23.3)	0 (0.0)	3 (75.0)

NSIP: Nonspecific interstitial pneumonia. OP: Organising pneumonia. GCs: Glucocorticoids. IS: Immunosuppressive agent. MMF: Mycophenolate mofetil.

**Table 5:** Multivariate Regression Analysis.

	Univariable Analysis		Multivariable Analysis	
	OR (95% CI)	*p*	OR (95% CI)	*p*
Age, (per year)	1.02 (0.97–1.08)	0.418	1.04 (0.98–1.10)	0.456
Sex (male)	1.40 (0.45–4.33)	0.559	1.63 (0.45–5.93)	0.231
Daily Oral GCs Use				
Never	Ref.		Ref.	
>0–19 mg	2.62 (0.38–17.99)	0.326	3.36 (0.45–24.72)	0.233
≥20 mg	5.02 (0.99–25.34)	0.051	6.13 (1.17–32.21)	0.032
Smoking, ever	1.12 (0.36–3.42)	0.848	1.15 (0.31–4.22)	0.837

GCs: Glucocorticoids. Ref.: “Never” group was used as the reference category for Oral GCs Use. Age was entered as a continuous variable (per year). Female sex was used as reference categories.

## Data Availability

The data presented in this study are available from the corresponding author upon reasonable request. Because the data are not publicly available due to privacy or ethical restrictions.

## Supplementary Materials

The following supporting information can be downloaded at: https://www.mdpi.com/article/10.3390/diagnostics16020299/s1, Table S1: Demographic, clinical and laboratory characteristics of the patients based on the survival status.

## Abbreviations

The following abbreviations are used in this manuscript:

IPAF	Interstitial pneumonia with autoimmune features
ILD	Interstitial lung disease
CTD	Connective tissue disease
CTD-ILD	Connective tissue-related-Interstitial lung disease
IPF	Idiopathic pulmonary fibrosis
UIP	Usual interstitial pneumonia
OP	Organising pneumonia
NSIP	Nonspecific interstitial pneumonia
IIM	Idiopathic inflammatory myopathies
Chest-CT	Chest computed tomography
PFT	Pulmonary function test
DLCO	Diffusion capacity for carbon monoxide
FVC	Forced vital capacity
ANA	Antinuclear antibody
RF	Rheumatoid factor
ULN	Upper limit of normal
Anti-CCP	Anti-cyclic citrullinated peptide
LTOT	Long-Term Oxygen Therapy
AZA	Azathioprine
MMF	Mycophenolate mofetil
CYC	Cyclophosphamide
GCs	Glucocorticoids
IS	Immunosuppressive agent
