# Supplementary material for: Interstitial Pneumonia with Autoimmune Features from the Rheumatologists’ Perspective; Single Center Experience
Source: Diagnostics (Basel). 2026 Jan 17;16(2):299. doi: 10.3390/diagnostics16020299 (PMC12840511; doi:10.3390/diagnostics16020299)
Supplement: Supplementary file 1 [file diagnostics-16-00299-s001.zip › diagnostics-4055369-supplementary.pdf]

Supplementary Table S1. Demographic, clinical and laboratory characteristics of the patients based on the survival status

	Deceased patients n= 16	Survived patients n= 56	*p*
**Age at diagnosis, year, mean (SD)**	64.5 (9.5)	62.2 (10.6)	0.423
**Follow-up duration, months, median (IQR)**	26.6 (48.9)	30.9 (56.7)	0.860
**Symptom Duration, years, mean (SD)**	2.0 (1.9)	2.4 (2.6)	0.615
**Female sex, n (%)**	9 (56.3)	36 (64.3)	0.558
**Smoking, ever, n (%)**	7 (43.8)	23 (41.1)	0.848
**BMI, mean (SD)**	28.0 (4.9)	29.8 (4.4)	0.232
**Comorbidities, n (%)**			
**Diabetes mellitus**	7 (43.8)	12 (21.4)	0.107
**Hypertension**	6 (37.5)	24 (42.9)	0.701
**Chronic lung disease**	1 (6.3)	9 (16.1)	0.440
**Atherosclerotic heart disease**	3 (18.8)	13 (23.2)	≥0.999
**Malignancy**	2 (12.5)	4 (7.1)	0.609
**LTOT, n (%)**	8 (50.0)	39 (33.9)	0.242
**Hypoxemia, n (%)**	5 (41.7)	9 (23.1)	0.272
**FVC <60%, n (%)**	4 (36.4)	9 (20.9)	0.429
**DLCO <60%, n (%)**	7 (77.8)	28 (73.7)	≥0.99
**CRP, n (%)**			0.381
**Normal**	6 (37.5)	25 (44.6)	
**≥2xULN**	8 (50.0)	18 (32.1)	
**ESR, median (IQR)**	25 (17.0)	16 (33.0)	0.581
**ANA, n (%)**			0.778
**Low positive ANA**	3 (18.8)	18 (32.1)	
**ANA ≥320 (diffuse, speckled, homogenous)**	5 (31.3)	15 (26.8)	
**ANA positivity (nucleolar or centromere)**	4 (25.0)	12 (21.4)	
**RF > 2xULN, n (%)**	2 (13.3)	14 (25.9)	0.492
**Anti-CCP >ULN, n (%)**	2 (16.7)	10 (20.0)	≥0.999
**Anti-dsDNA, n (%)**	2 (15.4)	5 (10.9)	0.643
**Anti-Ro (SS-A), n (%)**	0 (0.0)	5 (9.4)	0.583
**Anti-La (SS-B), n (%)**	1 (6.3)	4 (7.5)	≥0.999
**Ro-52, n (%)**	2 (12.5)	3 (5.7)	0.328
**Anti-Smith, n (%)**	0 (0.0)	2 (3.8)	≥0.999
**Anti-Pm/Scl, n (%)**	0 (0.0)	4 (8.0)	0.565
**NSIP, n (%)**	14 (87.5)	46 (82.1)	≥0.999
**Treatments, n (%)**			
**Pulse Glucocorticoids**	2 (12.5)	1 (1.8)	0.122
**Oral GCs ever**	14 (87.5)	35 (62.5)	0.059
**Daily Oral GCs dose**			0.088
**Never**	2 (12.5)	21 (37.5)	
**>0-19 mg**	3 (18.8)	12 (21.4)	
**≥20 mg**	11 (68.8)	23 (41.1)	
**IS without GCs**	0 (0.0)	6 (10.7)	0.327
**Cyclophosphamide**	0 (0.0)	3 (5.4)	≥0.999
**Methotrexate**	1 (6.3)	2 (3.6)	0.535
**Hydroxychloroquine**	1 (6.3)	5 (8.9)	≥0.999
**Rituximab**	1 (6.3)	1 (1.8)	0.397
**Mycophenolate mofetil**	1 (6.3)	7 (12.5)	0.674

**Azathiopyrine**	3 (18.8)	12 (21.4)	≥0.999
**Nintedanib/Pirfenidone**	1 (6.3)	2 (3.6)	0.535
**No treatment**	2 (12.5)	15 (26.8)	0.327

CRP: C- reactive protein. ESR: Erythrocyte sedimentation rate. GCs: Glucocorticoids. ULN: Upper limit of normal. LTOT: Long-term oxygen therapy. ANA: Anti-nuclear antibody. RF: Rheumatoid factor. Anti-CCP: Anti-cyclic citrullinated peptides.IS: Immunosuppressive agent. NSIP: Nonspecific interstitial Pneumonia
